# Plasmonic Optical Fiber-Grating Immunosensing: A Review

**DOI:** 10.3390/s17122732

**Published:** 2017-11-26

**Authors:** Tuan Guo, Álvaro González-Vila, Médéric Loyez, Christophe Caucheteur

**Affiliations:** 1 Institute of Photonics Technology, Jinan University, Guangzhou 510632, China; tuanguo@jnu.edu.cn; 2 Electromagnetism and Telecommunication Department, University of Mons, Boulevard Dolez 31, 7000 Mons, Belgium; alvaro.gonzalezvila@umons.ac.be (Á.G.-V.); mederic.loyez@umons.ac.be (M.L.)

**Keywords:** optical fibers, fiber Bragg gratings, plasmonics, sensing, nanoparticles

## Abstract

Plasmonic immunosensors are usually made of a noble metal (in the form of a film or nanoparticles) on which bioreceptors are grafted to sense analytes based on the antibody/antigen or other affinity mechanism. Optical fiber configurations are a miniaturized counterpart to the bulky Kretschmann prism and allow easy light injection and remote operation. To excite a surface plasmon (SP), the core-guided light is locally outcoupled. Unclad optical fibers were the first configurations reported to this end. Among the different architectures able to bring light in contact with the surrounding medium, a great quantity of research is today being conducted on metal-coated fiber gratings photo-imprinted in the fiber core, as they provide modal features that enable SP generation at any wavelength, especially in the telecommunication window. They are perfectly suited for use with cost-effective high-resolution interrogators, allowing both a high sensitivity and a low limit of detection to be reached in immunosensing. This paper will review recent progress made in this field with different kinds of gratings: uniform, tilted and eccentric short-period gratings as well as long-period fiber gratings. Practical cases will be reported, showing that such sensors can be used in very small volumes of analytes and even possibly applied to in vivo diagnosis.

## 1. Introduction

Biosensors bring a solution to the demand for direct, accurate and in situ monitoring in numerous fields such as genomics, proteomics, medical diagnosis, environmental monitoring, food analysis and security. Label-free optical biosensors enable real-time and direct observation of molecular interactions without using labels, since they sense binding-induced refractive index changes. To this aim, they usually combine a biological receptor compound and a physical or physicochemical transducer. Optical methods of transduction are usually minimally invasive, safe and provide multi-dimensional detection based on wavelength, intensity, phase or polarization metrology. They rely on well-established technologies (light sources, detectors, etc.) available from both telecommunication and micro-nano technologies industries, at optical frequencies in the visible and near-infrared regions. These ranges also coincide with a wide range of physical properties of bio-related materials, which is therefore perfectly suited for effective interrogation. 

**Figure 1:**
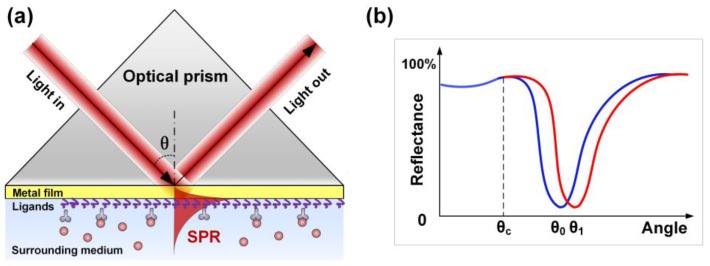
(**a**) Sketch of the Kretschmann prism configuration used for plasmonic sensing; and (**b**) its response to surrounding refractive index changes linked to biomolecules binding.

Within the available biosensor configurations (based on absorbance, reflectance, fluorescence, refractive index changes, among others), plasmonic devices combining dielectric and metal interfaces are particularly attractive. The strong sensitivity of the plasmon (oscillation of electrons at the metal–dielectric interface) propagation constant to the permittivity of bioreceptors grafted on the metal surface enables the detection and quantification of biochemical changes resulting from molecules binding. Practically, biochemical reactions are therefore measured by monitoring the effective refractive index shift of the so-called surface plasmon resonance (SPR). In a standard bulk approach, this is achieved with the Kretschmann prism that couples light beams from a glass medium to a thin metal layer above the critical angle so that light is totally reflected, as sketched in Figure 1. In this condition, an evanescent wave extends in both the metal layer and the surrounding dielectric medium, with a penetration depth not exceeding a fraction of the light wavelength (λ) [1]. Under phase-matching conditions [2,3], part of the light couples to the plasmon, which decreases the reflection at a given angle (*θ*_0_ in Figure 1, where *θ_c_* denotes the critical angle of incidence). This device is usually interrogated either by varying the wavelength and keeping the incidence angle constant or by using monochromatic light and modifying the angle. In both cases, the polarization state of the light is set parallel to the incidence plane so that the plasmon wave is orthogonally polarized with respect to the interface. Biochemical reactions happening at the surrounding metal interface slightly modify the effective refractive index of the plasmon wave, which is measured through an SPR shift (corresponding to an angle change from *θ*_0_ to *θ*_1_, as depicted in Figure 1). Such sensors present a surrounding refractive index (SRI) sensitivity of the order of 10^−6^–10^−7^ RIU (refractive index unit) [1,2,3,4].

While the Kretschmann prism configuration remains the most used today in commercial systems, optical fiber-based sensors bring numerous assets and are, therefore, the subject of intense research and development efforts. With their compactness and ease of connection, they provide remote operation in microliter volumes (or even below) of analytes and appear perfectly suited for in situ, or even possibly in vivo, diagnosis. In diagnosis, they can also assay different parameters simultaneously, either in line (sensing regions multiplexed along a single optical fiber) or in parallel, through the use of different fibers connected to a single read-out device. 

To excite SPR on the side of an optical fiber, a physical access to the core-guided light is locally required so that it can be brought into contact with the surrounding medium. A straightforward configuration for doing this is fiber bending [5,6]. Other solutions expose the core to the surrounding medium through a polishing or etching of the cladding, totally or in part [7,8,9,10,11]. The first reported configuration is based on this mechanism and dates back to 1993 [12]. More advanced configurations make use of fiber gratings permanently photo-inscribed in the fiber core that enable light coupling to the surrounding medium. Metal-coated fiber gratings have been attracting ever-growing interest over the years, as they bring specific practical benefits such as: (1) tuning of the SPR mode-excitation wavelength via selective cladding mode coupling; (2) subsequent compatibility with telecommunication-grade optical fibers and equipment; (3) temperature self-compensation; (4) fiber integrity conservation; and (5) compatibility with mass production, among others. Hence, numerous developments are nowadays obtained with this technology since it enables SPR excitation in the telecommunication wavelength window of around 1550 nm. This article will describe their operating principle and review the main achievements obtained for sensing selective proteins and cells with such devices. It will focus on recent progress in this field, which slowly but surely paves the way towards the use of these devices for minimally-invasive diagnosis.

## 2. Review of Grating Configurations Used for SPR Excitation

Gratings are usually manufactured in single-mode optical fibers, made of an 8 µm thick core surrounded by a 125 µm cladding. Such fibers are widely available at low cost (less than 100 US$ per km) and are telecommunication-grade. Overall, gratings preserve the fiber integrity (only the polymer jacket is removed at the grating location) while providing a strong coupling between the core-guided light and the cladding. Different grating configurations in single-mode optical fibers can be used for SPR excitation. Their characteristics and operating principle will be summarized hereafter. 

### 2.1. Unclad Uniform Fiber Bragg Gratings

**Figure 2:**
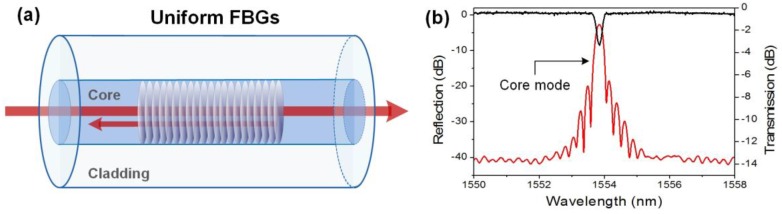
(**a**) Sketch of the light mode coupling in a uniform fiber Bragg grating (FBG); and (**b**) transmitted (black curve)/reflected (red curve) amplitude spectra of a 1 cm long uniform FBG.

A uniform fiber Bragg grating (FBG) is a periodic and permanent refractive index modulation of the fiber core with fringes perpendicular to the propagation axis [13,14]. It behaves as a selective mirror in wavelength for the light propagating in the core, reflecting a narrow spectral band centered on the so-called Bragg wavelength, as depicted in Figure 2. According to the phase-matching condition, the latter is given by λ_Bragg_ = 2n_eff,core_Λ where n_eff,core_ is the effective refractive index of the core mode (close to the refractive index of silica, 1.45 at 1550 nm), and Λ is the grating period. Most often, Λ is ~530 nm to ensure that the Bragg wavelength falls in the band of minimum attenuation of the optical fiber centered on 1550 nm, but gratings can be made at almost any wavelength also in the visible domain [15,16]. This is especially true relying on the point-by-point (or line-by-line) inscription process with a femtosecond pulses laser [17,18]. The most common means of production remain the phase-mask technique [19] and interferometric methods (both Talbot interferometer [20] and Lloyd mirror [21] configurations), most often used with a continuous frequency-doubled Argon laser emitting at 244 nm or a pulsed excimer laser emitting at 248 nm (ArF) or 193 nm (KrF). 

The Bragg wavelength is inherently sensitive to temperature and axial strain, through a change of both n_eff_ and Λ [22]. In practice, a change of temperature of +1 °C yields a Bragg wavelength shift of ~10 pm at 1550 nm. The sensitivity to axial strain is of the order of 1.2 pm/µε, also at 1550 nm. These sensitivities decrease with the operating wavelength. Such changes are easily measured with standard telecommunication instruments since the full spectral width of the main reflection band from a typical 1 cm-long grating is of the order of 100 pm.

As it corresponds to light confined in the fiber core, the Bragg resonance is not directly suited to excite a surface plasmon wave at the metal-surrounding medium interface. Hence, in practice, unclad optical fibers are used, with the core exposed to the surrounding medium so that an evanescent wave can extend in this medium. This is usually obtained through a chemical etching process, most often with hydrofluoric acid (HF) [23,24,25,26,27,28,29,30] or through a side-polishing process [31,32,33,34,35]. The latter configuration has strong similarities with the use of Bragg gratings produced in D-shaped optical fibers [36].

Unclad FBGs can be used to sense SRI changes in the range [1.30–1.45]. The maximum SRI sensitivity is obtained for values above 1.40, since the effective refractive index of the core mode is close to 1.447 at 1550 nm for a standard single-mode optical fiber. An SRI change yields a Bragg wavelength shift in the amplitude spectrum of the grating.

### 2.2. Tilted-Fiber Bragg Gratings

**Figure 3:**
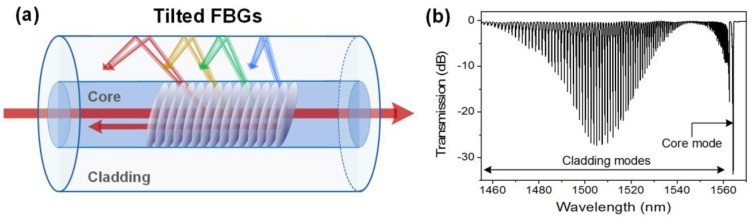
(**a**) Sketch of the light mode coupling in a tilted-fiber Bragg grating (TFBG); and (**b**) transmitted amplitude spectrum of a 1 cm-long 10° TFBG.

Tilted-fiber Bragg gratings (TFBGs) are short period (~500 nm) gratings with a refractive index modulation angled (*θ* < 45°) with respect to the perpendicular to the optical fiber axis [37]. In addition to the self-backward coupling of the core mode at the Bragg wavelength, TFBGs redirect some light to the cladding. There, the diameter is such that several possible cladding modes can propagate, each with its own phase velocity (and, subsequently, effective refractive index n_eff,clad_) [37]. These possible modes of propagation correspond to different ray angles, as sketched in Figure 3. There is a one-to-one relationship between the wavelength at which coupling occurs for a given cladding mode and its effective refractive index. This relationship is expressed by a similar phase-matching condition as for uniform FBGs: λ^i^_clad_ = (n_eff,core_ + n^i^_eff,clad_)Λ where the superscript i denotes the mode number. Figure 3 displays the transmitted amplitude spectrum of a 1 cm-long 10° TFBG. Each resonance of the spectral comb corresponds to the coupling from the core mode to a group of backward-propagating cladding modes. As a result of phase matching, the spectral position of a resonance depends on the effective refractive index of the corresponding cladding mode, which in turn depends on the optical properties of the medium over or near the cladding surface.

Therefore, spectral shifts of individual resonances can be used for refractometry purposes, either in the surrounding medium or inside a thin coating deposited on the fiber outer surface. The first demonstration of SRI sensing with TFBGs dates back to 2001 [38]. For an SRI increase between 1.30 and 1.45, a progressive smoothing of the transmitted amplitude spectrum was reported, starting from the shortest wavelengths. In practice, two main demodulation techniques can be used to correlate the spectral content with the SRI value quantitatively. The first method considers the global spectral evolution and involves monitoring the area delimited by the cladding mode resonance spectrum, through a computation of the upper and lower envelopes as resonances gradually disappear when the SRI reaches the cut-off points of each cladding mode [38,39]. The other method is more local and tracks the wavelength shift and amplitude variation of individual cladding mode resonances as they approach the cut-off wavelength. The latter is the wavelength at which the effective refractive index of a given cladding mode resonance equals that of the surrounding medium [40]. Both techniques present minimum detectable SRI changes of ~10^−4^ RIU when used with bare gratings. In terms of wavelength shift, this yields a sensitivity that peaks between 10 nm/RIU and 25 nm/RIU for the modes near cut-off. In all cases, the Bragg wavelength provides an absolute power and wavelength reference, which can therefore be used to remove uncertainties related to systematic fluctuations (such as unwanted power-level changes from the light source) and even ambient temperature changes. Indeed, all cladding mode resonances shift similarly to the Bragg resonance when temperature varies, as reported in [41].

The higher the tilt angle value, the higher the coupling to higher-order cladding modes. For tilt angle values exceeding 30°, it is possible to couple light to modes with an effective refractive index close to 1.00, which can be used for refractometry in gaseous media [42,43].

### 2.3. Excessively Tilted Fiber Gratings

**Figure 4:**
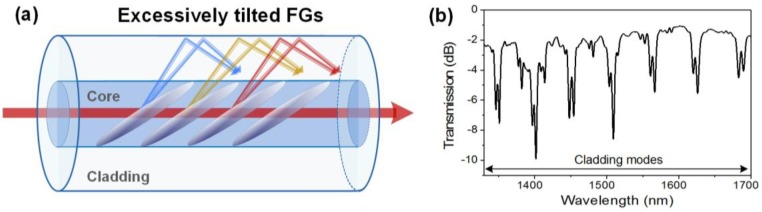
(**a**) Sketch of the light mode coupling in an excessively tilted fiber grating (ETFG); and (**b**) transmitted amplitude spectrum of a 1 cm-long ETFG.

Excessively tilted fiber gratings (ETFGs) are characterized by a tilt angle *θ* > 45° [44,45,46,47]. Given the very high angle value, they couple light to the cladding in the forward direction and present a hybrid behavior between the aforementioned weakly tilted FBGs and the long-period fiber gratings introduced in Section 2.5. As depicted in Figure 4, their transmitted amplitude spectrum is composed of multiple cladding mode resonances (with FWHM of a few nanometers, typically) spread over a wavelength range of a few hundred nanometers. They are usually produced with a continuous-wave laser using custom phase masks with a period higher than standard ones used for uniform and weakly tilted FBGs photo-inscription. Amplitude masks can also be used. In [48], theoretical considerations about light coupling in these structures are presented. 

### 2.4. Eccentric Fiber Bragg Gratings

**Figure 5:**
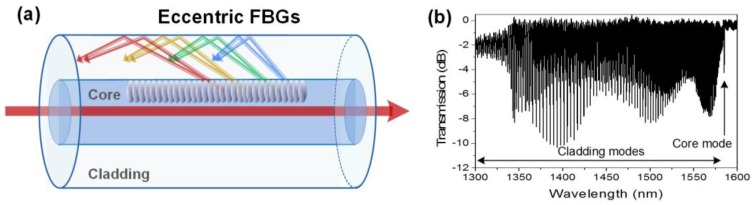
(**a**) Sketch of the light mode coupling in an eccentric fiber Bragg grating (EFBG); and (**b**) transmitted amplitude spectrum of a 1 cm long EFBG.

Eccentric fiber Bragg gratings (EFBGs) correspond to a point-by-point refractive index modulation highly localized in the core, close to the cladding region [49,50,51,52], as sketched in Figure 5. They are usually obtained with a tight focussing of a femtosecond pulses laser and the use of an air-bearing translation stage. Their strong localization close to the core-cladding interface induces light coupling in the fiber cladding, in a manner very similar to weakly tilted FBGs. Hence, their transmitted amplitude spectrum looks like a dense spectral comb, with hundreds of cladding mode resonances. An important difference with respect to TFBGs should be noted: EFBGs continuously couple cladding mode resonances with effective refractive indices ranging from 1.45 (at the right-hand side of the spectrum) to 1.00 (at the left-hand side) where they are cut-off when the grating is surrounded by air [53]. In the case of TFBGs, light coupling happens in privileged wavelength ranges, depending on the tilt angle value [43]. Their sensitivity to surrounding refractive index changes is very similar to that of TFBGs [50]. 

### 2.5. Long-Period Fiber Gratings

**Figure 6:**
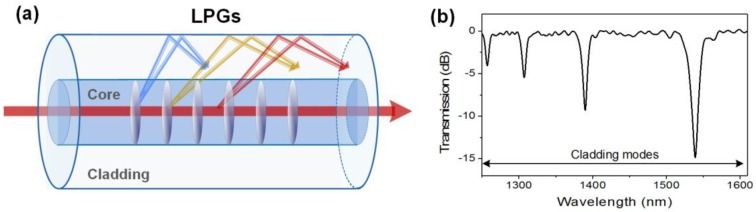
(**a**) Sketch of the light mode coupling in a long-period fiber grating (LPFG); and (**b**) transmitted amplitude spectrum of a 1 cm-long LPFG.

Long-period fiber gratings (LPFGs) correspond to a periodic refractive index modulation of the fiber core with a uniform period of a few hundreds of µm. In single-mode optical fibers, they couple the forward-going core mode into forward-going cladding modes [54,55], as illustrated in Figure 6. Their transmitted amplitude spectrum is composed of a few wide resonances (FWHM ~ 20 nm) spread over a wavelength range of several hundreds of nm. These resonances appear at wavelengths given by the following phase matching condition: λ^i^_clad_ = (n_eff,core_ − n^i^_eff,clad_)Λ. As they propagate close to the cladding-surrounding medium interface, these modes are inherently sensitive to SRI changes, which yield important wavelength shift [56,57,58]. They are also sensitive to bending so that care must be taken to avoid unwanted spectral fluctuations when LPFGs are used for SRI sensing [59]. LPFGs are usually produced through amplitude masks. They can also be easily manufactured point-by-point or using electric arc discharges.

### 2.6. Additional Considerations

SPR optical fiber sensors can be derived from the aforementioned grating structures surrounded by a thin metal film (most often gold or silver). Sheaths of thickness ranging between 30 nm and 70 nm or nanoparticle arrays are generally used. It is known that continuous metal films yield SPR generation, spectrally manifested by the coupling between a given cladding mode resonance and the SPR mode. Nanoparticles usually excite localized SPR (LSPR), corresponding to a broader attenuation of numerous cladding modes [60,61,62]. In surface refractometry, they can offer a comparable sensitivity to that of SPR-based sensors. The properties of the metal nanoparticles are highly dependent on their constitutive material, also in shape and size. Hence, controlling these three parameters allows to optimize the performances for detecting a particular target [63]. 

**Figure 7:**
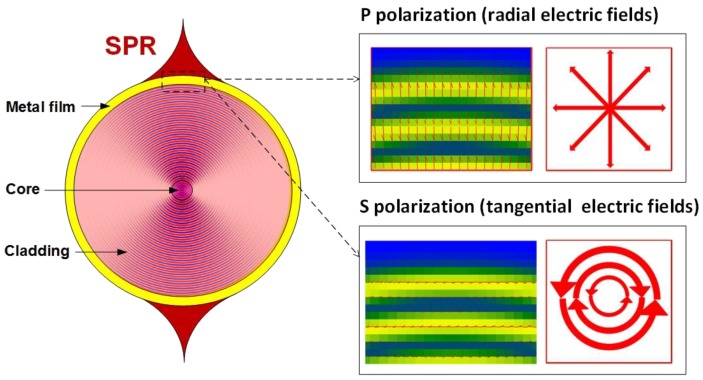
Sketch of the surface plasmon resonance (SPR) excitation around an optical fiber showing the required polarization of the electric field.

As depicted in Figure 7, SPR generation is achieved when the electric field of the light modes is polarized mostly radially at the surrounding medium interface. The orthogonal polarization state is not able to excite the SPR, as the electric field of the light modes is polarized mostly azimuthally (i.e. tangentially to the metal) at the surrounding medium interface, and thus cannot couple energy to the surface plasmon waves. The SP phenomenon drastically enhances the sensitivity to surrounding refractive index changes at the outer surface of the optical fiber. Depending on the configuration, impressive refractometric sensitivities in the range [10^2^–10^4^ nm/RIU] have been reported [11,64]. Very recently, an SRI sensitivity even exceeding 10,000 nm/RIU has been theoretically reported for plasmon-assisted excessively tilted gratings [65].

It is worth noting that when comparing the sensor performances between different configurations, especially for immunosensing where different parameters (surface covering, antibody/antigen affinity, etc.) come into play, it is not sufficient to compare only sensitivities (i.e., wavelength shifts), without considering the wavelength-measurement accuracy. In practice, it is more convenient to refer to the figure of merit (FOM) of the device, which corresponds to the ratio between the sensitivity and the linewidth of the resonance. A narrow resonance can be measured with a high resolution so that its exact location can be computed, which is not true for a broad one [66]. As a result, in terms of experimentally demonstrated FOM, because gratings usually feature narrow cladding mode resonances (except for LPFGs), they outperform all other optical fiber configurations [11]. However, the goal of this paper is not to compare the relative performances of the grating architectures compared to other optical fiber configurations. Readers interested in additional considerations about sensing configuration and performance are encouraged to consult these complementary review papers [64,67]. 

## 3. Interactions with Metals and Surface Biochemical Functionalization

### 3.1. Metal Layer Deposition

**Figure 8:**
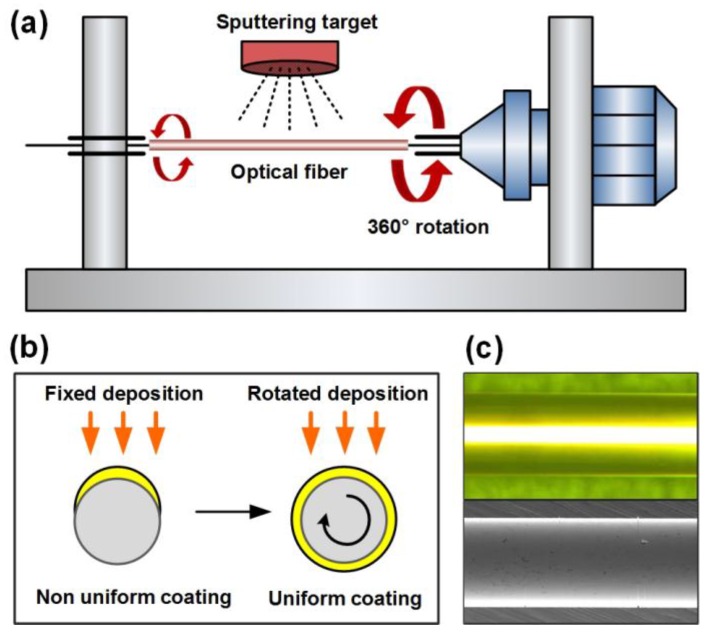
(**a**) Sketch of the sputtering (or vacuum evaporation) deposition process to obtain a uniform metal thickness all around the optical fiber cross-section; (**b**) principle of the double deposition process; and (**c**) microscope view of a gold-coated fiber surface.

The shape of deposited metal particles and the coating homogeneity directly impact the sensitivity and the reproducibility of plasmonic sensors. Metal deposits can be successfully applied to optical fibers using well-established technologies, such as nanoparticles immobilization [68], nanostructures elaboration [69], electroless deposition [70], evaporation or sputtering [71]. The latter is the most spread technique for planar covering, because it provides very high quality depositions. However, in case of cylindrical surfaces, as for optical fibers, two consecutive depositions are usually performed in the same conditions, with a 180° rotation between both processes to ensure an entire surface covering. More sophisticated devices may allow a continuous rotation during the sputtering process, providing a more uniform coverage, as sketched in Figure 8.

To enhance the adherence of gold on silica substrates, different methods have been investigated, such as the deposition of 2–3 nm of chromium or titanium under the Au layer [72], ion beam bombardment [73] and finally, the use of polymers and adhesive molecules [74]. Another option consists in thermally annealing the gold film, modifying its morphology [75] and consequently, the sensor’s potential of detection [76].

Regardless of the chosen deposition technique, the main challenge is to obtain a very uniform layer where thickness, rugosity and morphology are finely managed for an optimum SPR excitation. A gold thickness of about 50 nm is needed to get the narrowest and the deepest SPR attenuation, corresponding to the highest surface sensitivity of the sensor. Recent papers also mention the use of non-metal layers for SPR analysis, such as semiconductors and oxides [77] but these kinds of materials have to be explored for optical fiber sensors and represent interesting perspectives for the future. 

### 3.2. Surface Biofunctionalization

Metal-coated fiber gratings are then functionalized for biosensing purposes. The chemistry involved for the surface activation depends on the target application. For example, numerous biosensors use the antibody/antigen affinity to detect proteins of interest, but a large panel of variants exist, using for example DNA hybridization [78,79], enzymes and biomimetism (phage display and recombinant protein engineering, aptamers technologies, oxidative state sensing) [80]. New emerging technologies are also increasingly used to expand the target possibilities and enhance the sensor response, such as the immobilization of nanobodies, affimers or estrogens [80,81,82,83].

**Figure 9:**
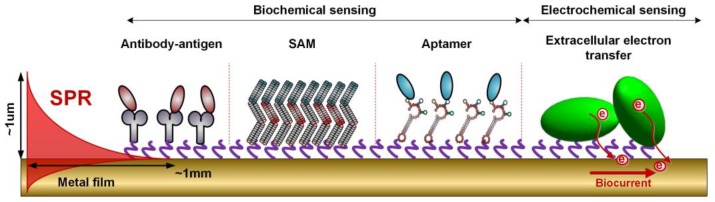
Sketch of a typically biofunctionalized metal-coated optical fiber surface, showing the different strategies that are most often used to attract analytes.

Concerning the analyzed parameters, the biosensing area can be used to detect the presence of target analytes such as biomarkers for clinical measurements, but also to monitor cellular behaviour and densities, the development of biofilms, the detection of bacteria and viruses, environmental monitoring, etc. Whatever the analyte to be detected, the most common technique for immobilizing recognition molecules on the sensor is the elaboration of a self-assembled monolayer (SAM) [84]. For this, metal-coated optical fibers are first cleaned with absolute ethanol and immersed in a thiols solution. Practically, this can be done in a capillary tube sealed at both ends after the insertion of the fiber, to prevent solvent evaporation. After this, the functionalized fiber gratings are rinsed again and the surface is activated using the selected biomolecules. An effective blocking step is also needed after the functionalization to ensure a high specificity and a low rate of false positive responses when analyzing complex media. Generally, this yields the structure depicted in Figure 9.

## 4. Interrogation of Plasmonic Fiber-Grating (Bio)chemical Sensors

**Figure 10:**
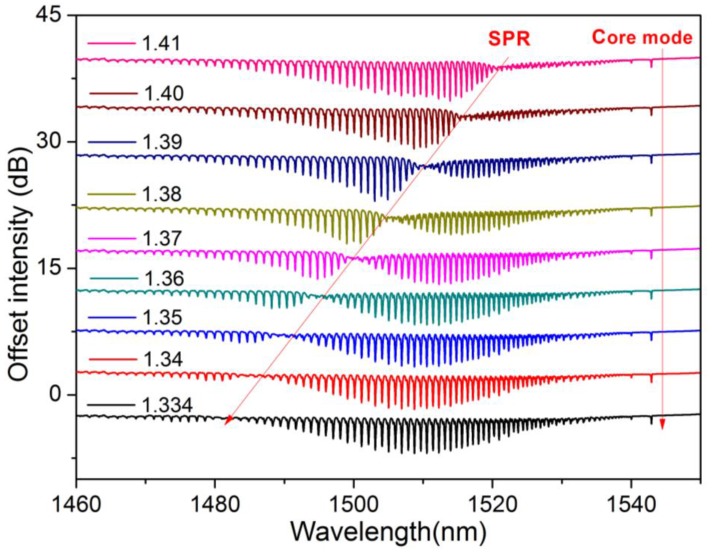
Evolution of the SPR mode in the transmitted amplitude spectrum of a gold-coated 10° TFBG as a function of a change of the surrounding refractive index.

In the case of plasmonic optical fiber grating-based configurations, the refractive index variations are reflected on the optical spectrum in the form of both a wavelength shift and an optical power change [85,86,87], as depicted in Figure 10 for a gold-coated TFBG. 

Bioreceptors are functionalized on top of the metallic coating [88] and the spectral modifications result from the molecular interactions happening on this outer film. Several ways of interrogating these sensors co-exist and the application of one or the other mostly depends on the requirements of the specific application. Spectral and intensity interrogation are the two main techniques widely reported, and an advantage of utmost importance with regard to other technologies is their implementation with commercial optical-fiber equipment or conventional FBG interrogators. Other techniques exist as well and have proved to exhibit even better resolution, but their applications are limited to laboratory environments due to their complexity.

### 4.1. Spectrometer-Based Interrogation

**Figure 11:**
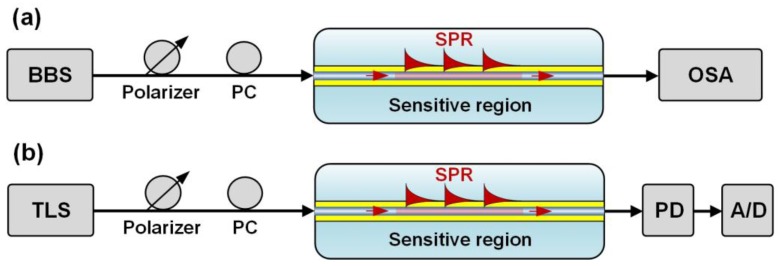
Scheme of the classic implementations of (**a**) spectral, and (**b**) intensity interrogation, of the sensors in transmission.

This technique relies on the quantification of the wavelength shift induced by a refractive index change in the sensing region [89]. It has been the most widespread method for interrogating plasmonic FBG sensors since the early stages of this technology, and hence the majority of commercially available FBG interrogators are based on the same principle. As can be seen in Figure 11a, the classic implementation consists of a broadband source (BBS) and an optical spectrum analyzer (OSA) for interrogating the transmission response of the sensors. In the case of etched FBGs [90,91] and LPFGs [92,93,94], the wavelength tracking can be carried out in a wider region than the one required for TFBGs [95,96,97,98,99] and eccentric FBGs [49], since for these configurations the most sensitive cladding mode must be carefully isolated from the rest.

For sensor interrogation in reflection mode, an optical fiber circulator is located between the source and the sensor. The OSA is also connected so that both source and detector remain located at the same side of the optical path. For etched FBGs no additional element is required due to the nature of their reflection response [100] and certain algorithms can be used for increasing the resolution of the measurements [101]. For the rest of the summarized alternatives, a mirror is deposited on the fiber tip in order to allow back-reflection of the light modes that are sensitive to the external medium [102]. Practically, some FBG interrogators integrate the source, circulator and detector as one, so that the sensor can be connected to a single port of the device. In this precise case, the source can be a tunable laser, offering a very high wavelength resolution (1 pm typically) and a fast acquisition rate (most often in the range 1–10 Hz with currently available commercial FBG interrogators) over the C + L wavelength bands.

### 4.2. Intensity or Optical Power-Based Interrogation

Another method to interrogate plasmonic FBG-based sensors is the quantization of the intensity variations produced as a result of the coupling of light to the plasmon wave. The most typical configuration for an interrogation of the transmitted response is that shown in Figure 11b. The interrogation is carried out in a narrow band of the optical spectrum, so a tunable laser (TLS) can be used as a source, together with a photodiode (PD) as detector and an analog-to-digital converter (A/D) to obtain the desired data [41,103]. The function of the TLS is matching the wavelength of the most sensitive mode of the fiber grating, so once the sensor is characterized it can be replaced by a common laser. This technique relies on the principle of edge filtering [104] so that the optical power change is produced as a result of the wavelength shift of the mode with respect to the fixed wavelength of the laser source. Several cost-effective configurations have been reported for interrogating both the transmission and reflection response of the sensors, mainly based on different associations of fiber gratings to filter the spectral region of interest [105,106,107]. It is also worth mentioning that the principle of intensity interrogation has recently been applied for the development of reduced size plasmonic optical fiber sensors interrogated with the flashlight and camera of a common smartphone [108], although a fiber grating-based counterpart is yet to be investigated.

### 4.3. Other Interrogation Techniques

When working with plasmonic fiber-grating sensors, light needs to be properly polarized in order to obtain a good performance [109]. Additional interrogation techniques have been reported making use of polarization analysis to evaluate the sensor response with respect to the interactions produced in the surrounding medium. The measurement of the phase of the light at a wavelength matching the SPR [110] or the use of the polarization-dependent loss (PDL) [111] are two techniques that have been proven to exhibit a good performance, especially when associated with nanoparticles [112,113]. In the case of fiber gratings, the PDL spectrum relates to the difference between orthogonally polarized spectra [114], which therefore contains information about (L)SPR.

## 5. Protein and Cell Detection and Quantification

Since the first reports about the use of bare fiber gratings for biosensing [115,116], numerous experimental demonstrations, most often in complex media mimicking the final environment where the sensors should be used in practice, have confirmed that metal-coated fiber gratings can be successfully used for biochemical sensing. 

This section first summarizes prominent examples of biosensors based on metal-coated fiber gratings. Then, considering a practical study case where functionalized gratings are used for cancer diagnosis in tumors, Section 5.2 will outline the typical roadmap that needs to be followed to build such plasmonic optical fiber-grating immunosensors, ensuring their use for minimally-invasive diagnosis.

### 5.1. Overview of Plasmonic Fiber-Grating Immunosensors

In this section, a general survey of the recent literature in this topic is presented in Table 1 in which the main characteristics and performance indicators of experimental demonstrations are given. Hence, whenever possible, the limit of detection of the target molecules is specified. The latter strongly depends on the characteristics of the analytes, especially their nature and mass. So, making a fair comparison between the relative performances of these various immunosensing experiments is particularly tough and will not be covered by this article. Also, while non-metalized configurations have demonstrated very good performances for biosensing and immunosensing [117,118,119,120], this table focuses on grating configurations where metals are used, either in the form of sheath or nanoparticles, as specified in the dedicated table entry.

### 5.2. Detection of Cancer Biomarkers

This subsection summarizes the standard roadmap usually pursued to build plasmonic fiber-grating immunosensors. It shows the generic process, on the one hand, and focuses on a practical example linked to a relevant clinical issue, on the other hand: the minimally invasive detection of cancer biomarkers. 

In [88,102], the focus was made on cytokeratins (both CK7 and CK17) that are usually used for diagnosis in oncology, especially in the differential diagnosis of lung carcinomas, since primary tumors express CK7 while secondary tumors are deficient. Moreover, it has been demonstrated that cytokeratin fragments can be released from malignant cells and can reach the blood circulation. They are, therefore, easily accessible with an optical fiber that has been properly modified.

Sensors prototyped for minimally invasive diagnosis, as those reported in [88,102], can be prepared following the main steps listed in Table 2 below.

**Figure 12:**
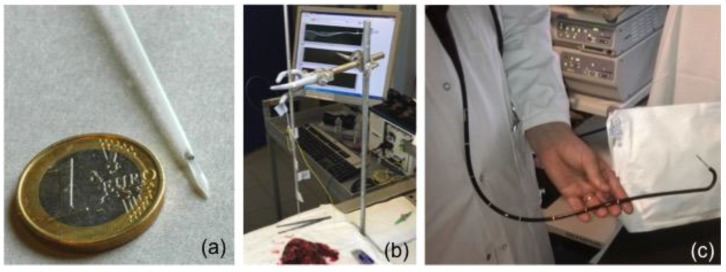
(**a**) Picture of the packaged plasmonic fiber-grating sensor; (**b**) biosensor inserted in a freshly biopsied tissue and its corresponding amplitude spectrum; and (**c**) packaged sensor inserted in the operating channel of an endoscope.

Depending on the applications, these steps can be complemented with:

(1) Mirror deposition on the fiber cross-section to operate in reflection mode. Optical fibers containing gratings are cleaved beyond the grating location to use them in reflection, through the use of a silver mirror deposited on the cleaved fiber end face. This can be as simple as using a silver glue.
(2) Grating insertion into a specially-designed protective packaging allowing it to be used in various media. In [102], a packaging was made of a hollow cylindrical needle manufactured in polyoxymethylene C2521 Hostaform. As depicted in Figure 12, this packaging provides a window to expose the sensor location to the surrounding medium. It has been tailored for possible insertion in the operating channel of an endoscope.

The study conducted in [88,100] has demonstrated that these immunosensors reach a limit of detection of 1 pM in phosphate buffered saline (PBS) media supplemented with 10 % of serum. Then, the detection of cytokeratins trapped in the crosslinked polymer network of a porous gel matrix was reported, despite the non-liquid nature of the hosting medium. These results have allowed the sensors to be inserted in fresh tissues obtained from a biopsy. Such measurements have been conducted at the hospital and have demonstrated a positive biosensor response in tumoral tissues. 

These first results constitute an important milestone towards the possible demonstration of in vivo diagnosis using plasmonic fiber-grating biosensors. To this end, numerous developments and validation tests remain to be done, both in terms of extensive testing of the devices in different kinds of tumors and to fully demonstrate the biocompatibility and compatibility with standard practices in the sterilization of the probes. 

## 6. Conclusions

This review of recent developments in fiber grating-based SPR immunosensors confirms the increasing level of maturity obtained in the field, essentially resulting from the complementary efforts made by photonics and biochemistry groups all around the world. This development comes along with the use of more sophisticated experimental protocols and more realistic error analyses. We can now assert that grating-based SPR sensor platforms have passed the proof-of-principle level, as they have been tested in complex media that replicate the final application environment. Grating-based SPR sensors can operate easily in the mid-infrared wavelength range, via the resonant coupling between the cladding modes and the surface plasmon wave. Hence, they are compatible with the use of cost-effective and high-resolution FBG interrogators that can be easily computer-driven to process the spectral information in real time. Most grating architectures also provide a response that is inherently immune to ambient temperature fluctuations, which is highly desirable for practical implementation. 

Of course, in addition to work on relevant clinical applications, some new developments also offer exciting possibilities for future research, notably in the areas of microstructured fibers [132] and plastic optical fibers—that are inherently much more biocompatible than their silica counterparts—[133,134] or even the use of natural fibers [135]. Another important path of exploration is the realization of nanostructures in metals [136] or the inclusion of carbon nanotubes, graphene and other novel plasmonic materials, such as oxides and nitrides, in sensor fabrication [77,137,138]. Recent work indicates that combining graphene with noble metal particles and layers promises a wealth of new physics and sensing modes, in addition to intrinsic tunable and adjustable plasmonic properties [91,139,140,141]. It is hoped that this review will help in fostering further research in the field of fiber-grating SPR biosensors.

## Figures and Tables

**Table 1:** Summary of the main characteristics of different plasmonic fiber-grating immunosensors reported so far.

Grating Architecture	Functional Materials	Analyte and Sensor Performances	Ref.
LPFG	SiO_2_:Au NPs modified with biotin	Streptavidin detection Sensitivity: 6.88 nm/(ng/mm^2^)	[121]
LPFG	Self-assembled Au colloids + dinitrophenyl compound (DNP)	Detection of anti-DNP LOD: 950 pM	[122]
TFBG	Au layer + thiol-modified aptamers	Thrombin detection in buffer and serum solutions LOD: 22 nM	[123,124]
TFBG	Au layer + self-assembled monolayer (SAM) + anti-transferrins	Transferrin detection LOD: 10^−6^ g/mL	[125]
TFBG	Au layer + fibronectin	Analysis of cellular behavior under different stimuli	[126]
TFBG	APTMS, glutaraldehyde and cysteamine thin films + Au nanocages/nanospheres	Biotin detection LOD: 11 pM (nanospheres)–8 pM (nanocages)	[68]
TFBG	Au layer + boronic acid	Glycoprotein detection LOD: 2 × 10^−5^ g/mL	[127]
TFBG	Au layer + SAM + anti-cytokeratins + bovine serum albumin (BSA)	Detection of cytokeratins 7 and 17 for lung cancer diagnosis LOD: 1 pM	[88,102]
TFBG	Au layer + SAM + EGFR (epidermal growth factor receptor) antibodies	Detection of epithelial cells through their EGFR LOD: 3 × 10^6^ cells/mL	[128]
TFBG	Au layer with different thicknesses	Detection of proteinuria in rat urine LOD: 1.5 × 10^−3^ mg/mL	[129]
TFBG	Au layer + SAM + aquaporin-2 antibodies	Detection of aquaporin-2 for nephrotic syndrome analysis LOD: 1.5 ng/mL	[130]
ETFG	Au NPs + cysteamine + activated staphylococcal protein A	Detection of Newcastle disease virus LOD: 25 pg/mL in a 200 µL volume	[97]
FBG	Oligonucleotide-functionalized Au NPs	DNA target sequences	[131]

**Table 2:** Main steps required to modify an optical-fiber section into a plasmonic fiber-grating immunosensor.

Stage	Generic Process	Practical Implementation in [88,102]
1. Grating manufacturing and optimization	– Local (mechanical or chemical) stripping of the polymer jacket of a photosensitive or hydrogen-loaded standard single mode fiber. – Use of dedicated laser and technique to photo-inscribe a grating in the stripped region. – Thermal annealing (in the case of hydrogen-loaded fibers) to stabilize the grating spectrum.	– 1 cm long 7° TFBGs in hydrogen-loaded standard telecommunication-grade single-mode optical fibers. – Use of a frequency-doubled argon laser emitting 60 mW at 244 nm and the phase mask technique. – Thermal annealing at 100 °C during 24 h.
2. Metal deposition and optimization	– Surface fiber cleaning with ethanol and/or piranha solution to remove contaminants. – Metal deposition using one of the techniques described in Section 3.1 (+use of a buffer layer or thermal annealing to improve gold adherence).	– ~50 nm gold coating deposited around the TFBGs using a sputtering process (thickness measured with a built-in Quartz microbalance). – Two depositions with 90° rotation between each. – Thermal annealing during 2 h at 200 °C. – Washing using absolute ethanol and dried under N_2_.
3. Biochemical functionalization	– Metal surface cleaning, usually with absolute ethanol. – Covalent immobilization of bioreceptors. This step strongly depends on the targets, as described in Section 3.2. – Deposition of blocking agents (most often bovine serum albumin (BSA) or milk caseins) to avoid unspecific interactions. – Rinsing to remove all non-linked molecules.	– Surface cleaning with absolute ethanol. – SAM of S_2_PEG_6_COOH alkanethiols on the gold surface. – Surface activation using NHS/EDC process. – Anchoring of anti-CK17 antibodies by immersion in a pH-controlled solution. – Deposition of BSA (5 % *w*/*v* in phosphate-buffered saline (PBS)). – Rinsing with PBS.
4. Interrogation and data-processing	– Splicing of the grating to fiber pigtails. – Connection to a measurement set-up including polarization control to record the reflected/transmitted amplitude spectrum (remote computer control for real-time operation). – Data-processing based on the tracking of the SPR mode as a function of time.	– Use of a MicronOptics FBG interrogator and a polarization controller, allowing to record spectral measurements at 10 Hz rate with 1 pm wavelength resolution). – Insertion of the sensors in various complex media (PBS + serum, gel matrices and fresh biopsied lung tissues). – Record of the amplitude spectrum and computation of the wavelength shift and amplitude variation of the most sensitive cladding mode resonance in the P-polarized spectrum.
